# Editorial: Abiotic and biotic stress in horticultural crops: insight into recent advances in the underlying tolerance mechanism

**DOI:** 10.3389/fpls.2023.1212982

**Published:** 2023-05-31

**Authors:** Milan Kumar Lal, Rahul Kumar Tiwari, Muhammad Ahsan Altaf, Awadhesh Kumar, Ravinder Kumar

**Affiliations:** ^1^ ICAR-Central Potato Research Institute, Shimla, Himachal Pradesh, India; ^2^ School of Horticulture, Hainan University, Haikou, China; ^3^ ICAR-Division of Crop Physiology and Biochemistry, National Rice Research Institute, Cuttack, Odisha, India

**Keywords:** horticultural crops, abiotic stress responses, biotic stress tolerance, pathogen, insect and nematodes, systemic resistance

## Introduction

The changing global climate and human activities have immensely impacted the production and productivity of horticultural crops (Varshney et al., 2018). The abiotic stress condition has led to various environmental restrictions such as salinity, sodic alkaline, drought, temperature fluctuations, and heavy metal exposure (Tiwari et al., 2021; Devi et al., 2022; Lal et al., 2022; Chen et al.; Zheng et al.). These factors can significantly affect plant growth, yield, and quality. Similarly, biotic stresses, such as viruses, fungi, bacteria, insects, vectors, and nematodes, can severely damage the vigour and productivity of horticultural crops (Kumar et al., 2021; Lal et al., 2021; El-Sappah et al.; Sun et al.; Yadav et al.; Samal et al.). Abiotic and biotic stress in horticultural crops impacts seed germination, growth, reproduction (including seed formation, flowering, and fruiting), and eventual decline (El-Sappah et al.; Sun et al. Samal et al.). Hence, it is imperative to investigate horticultural crops’ physiological, biochemical, and molecular reactions to ascertain the impact of abiotic stresses and recognize potential resistance mechanisms and ameliorating approaches.

Various interventions have been implemented in horticultural crops to mitigate the negative effects of abiotic and biotic stresses on crop plant (Williamson et al., 2002; Sonmez et al., 2009). These include biostimulant chemicals, hormones, novel chemicals, and microorganisms, which have been shown to enhance crop plant tolerance and ultimately lead to increased yield in horticultural crops (Chen et al.; Jaiswal et al.). Recent studies have indicated that the presence of phytochemicals, secondary metabolites, and antimicrobial peptides can alleviate the negative impacts of both abiotic and biotic stresses through the augmentation of enzymatic and non-enzymatic antioxidants, phytohormonal interactions, activation of defense genes, and systemic resistance (Xia et al., 2020; Altaf et al., 2021; Altaf et al., 2022; Moosa et al.; Tiwari et al., 2022; Najeeb and Li; Rahman et al.). Understanding the effects of abiotic and biotic stresses on horticultural crops and the mechanisms involved in mitigating these stresses can significantly improve crop productivity and quality. In this Research Topic, researchers around the world provided various mitigation strategies and used various novel biostimulant chemicals, hormones, novel chemicals, and microorganisms, as well as phytochemicals, secondary metabolites, and antimicrobial peptides, can help develop new strategies to enhance the resilience of horticultural crops to environmental stresses.

The regulation of stress response involves a significant mechanism known as phytohormonal cross-talk. Researchers have utilized epigenomics, genomics, proteomics, and metabolomics methodologies to comprehend the reaction of horticultural crops toward abiotic stress (Hoekenga, 2014; Zargar et al., 2017; Mishra et al., 2022; Yadav et al.; Zheng et al.). Osmotic adjustments and reactive species signalling are two key mechanisms involved in the response of horticultural crops to abiotic and biotic stress. Transcriptional and translational regulation is essential in stress response in these crops (Zheng et al.). Researchers are also exploring the application of novel phytoprotectants to mitigate stress in horticultural crops (Khan et al., 2014; Arnao and Hernández-Ruiz, 2020; Devireddy et al., 2021). Additionally, there is increasing interest in understanding the mechanistic insights of Plant Growth Promoting Rhizobacteria (PGPR) in regulating stress conditions in horticultural crops. PGPR-mediated regulation has been shown to improve the tolerance of crops to abiotic and biotic stresses (Vaishnav et al., 2016; Abbas et al., 2019; Bharti and Barnawal, 2019). By exploring various avenues such as physiological and biochemical responses, signalling mechanisms and pathways, phytohormonal cross-talk, epigenomics, genomics, proteomics, and metabolomics approaches, osmotic adjustments, reactive species signalling, transcriptional and translational regulation, novel phytoprotectants, and PGPR-mediated regulation, a more comprehensive comprehension of horticultural crops’ reaction to abiotic and biotic stress can be attained (Vance, 2010; Qin et al., 2020). The discoveries above have the potential to facilitate the formulation of novel approaches aimed at augmenting crop durability and output.

## Studies addressing factors affecting abiotic stress responses in horticultural crops

The world is currently experiencing changes in the global climate, and with the world population increasing rapidly, there is an urgent need to increase agricultural productivity (Rosegrant and Cline, 2003; Devaux et al., 2020; Kumar et al., 2023). According to projections, a 70 percent increase in agricultural output will be necessary by mid-century to satisfy the needs of the expanding population (Ortiz-Bobea et al., 2021). Enhancing agricultural productivity is paramount in tackling the world’s population-escalating food requirements (Knoppers et al., 2008). Given the impacts of climate change, such as elevated temperatures and severe weather phenomena, it is crucial to identify sustainable and effective methods for augmenting agricultural productivity (Zheng et al.). Achieving the horticultural productivity goal necessitates a multifaceted strategy encompassing cutting-edge technologies, embracing sustainable agricultural methodologies, and advancingovel horticultural cultivars (Devaux et al., 2020; Onoja and Adione, 2020). Furthermore, governments and organizations must allocate resources towards research and development initiatives to tackle the horticultural industry’s various obstacles(Yadav et al.; Zulfiqar et al.; Samal et al.).

The role of phytohormone and growth regulator in enhancing horticultural crops can lead to sustainable agricultural growth, which might enhance tolerance against abiotic stress tolerance (Zheng et al.). For example, the use of nano-nutrient solution (NNS) was used to alleviate the detrimental effect of drought on tomato crops. The application of about 1% NNS showed improved shoot length, fresh and dry weight, number of leaves and flowers. It increased the content of leaf chlorophylls, carotenoids, total phenolics, total soluble sugars, and flavonoids of tomato crop. The concentration of 3% NNS was found to minimize electrolyte leakage, while 5% NNS application exhibited higher total free amino acids and minimum lipid peroxidation rate in leaves. The growth regulator NNS could be a promising environmentally safe agricultural technique for mitigating the negative effects of drought stress on crop growth and yield (Mubashir et al.). Similarly, another study by Ahmed et al. showed that using zinc oxide nanoparticles (ZnO NPs) can effectively enhance plant growth and production of Coriandrum sativum L plant during drought. The foliar application of 100 ppm ZnO NPs in *Coriandrum sativum* L can improve net photosynthesis, stomatal conductance, and transpiration rate, as well as increase chlorophyll content and reduce abscisic acid levels in drought-susceptible plants. The efficacy of ZnO NPs in inducing drought tolerance was revealed through principal component analysis. The findings suggest that ZnO NPs can be a promising strategy to mitigate the negative impact of drought stress on *Coriandrum sativum* L (Ahmed et al.). Cold stress is also reported to affect the Hami melon and its keeping quality. However, components such as chitosan treatment can reduce fruit softening and chilling injury in cold-stored Hami melon. The study by Zhang et al. revealed that chitosan treatment maintained high levels of starch and sucrose contents, regulated enzyme activities and gene expressions related to starch and sucrose metabolism, and reduced fruit softening and chilling injury (Zhang et al.).

Moreover, the review on the aspect of the role of bio-stimulator such as salicylic acid (SA) in horticultural revealed that the use of SA can lead to enhance productivity and reduce the negative effects of abiotic stress conditions by enhancing signaling molecules, antioxidants, osmolytes, and secondary metabolites, as well as regulate the expression of stress-related genes (Chen et al.). Pre-harvest foliar spraying of salicylic acid (SA) and post-harvest caraway oil coating was tested to reduce chilling injury (CI) during post-harvest storage of sweet pepper. The caraway oil showed antifungal activity against *Botrytis cinerea* mycelia. The lowest CI was obtained with 3 mM SA and 0.6% caraway oil treatment. The treatment resulted in a delay in weight loss and firmness, as well as changes in pH, TSS (total soluble solids), TA (total acidity), and color. Additionally, the treatment led to an increase in capsaicin content. Incorporating SA (3 mM) and caraway oil (0.6%) might be a practical solution to improve sweet pepper quality during storage (Hanaei et al.). Using carbon-rich materials such as biochar (BC) also showed significant benefits for horticultural systems. BC amendments to soil or growing media improve seedling growth, increase photosynthetic pigments, and enhance photosynthesis, improving crop productivity in *Citrus sinensis* crop (Zulfiqar et al.).

The abiotic stress in the horticultural crops leads to changes in the biochemical and molecular response, ultimately providing tolerance. The role of certain genes, such as the overexpression of CpCHS1 gene from sweet cherry in tobacco has been shown to improve the germination frequency and fresh weight of transgenic seedlings under drought stress by enhancing the activity of SOD, POD, CAT, and proline under drought stress. These findings suggest that chalcone synthase plays a crucial role in regulating plant growth, development, and abiotic stress tolerance in sweet cherry and other Rosaceae species, which might have potential applications in improving the productivity and stress tolerance of cherry and other fruit trees (Hou et al.). Similarly, the overexpression of HSF20 in ‘Benihoppe’ strawberries made fruits sensitive to temperature, while overexpression of CBF/NF-Y promoted coloring under cold treatment. On the contrary, different temperatures affected hormone metabolism, anthocyanin, reactive oxygen species, and synthesis of terpenoids, amino acids, and phenylpropanoids, leading to changes in fruit quality and this aforementioned study provides a basis for further research on improving post-harvest quality of strawberries (Zheng et al.). The transcriptome analysis in *Brassica napus* revealed total of 79,061 unigenes, with 3,703 differentially expressed genes (DEGs) under low-temperature stress. The DEGs under low temperature stress revealed that the gene related to sugar metabolism, antioxidant defense system, plant hormone signal transduction, and photosynthesis was expressed (Hussain et al.). Similarly, the cold stress tolerance was enhanced in cucumber when figleaf was grafted on it, which leads to the activation of the WRKY41/WRKY46-miR396b-5p-TPR module by abscisic acid (ABA). The findings suggest that ABA-mediated figleaf gourd grafting-induced cold tolerance in cucumber plants is through activating the WRKY41/WRKY46-miR396b-5p-TPR module (Sun et al.). Another study by Hussain et al. in soybean highlights the importance of miRNA for low-temperature stress. It also sheds light on the role of miRNAs, such as miR319, miR394, miR397, and miR398, in regulating gene expression cold stress tolerance (Hussain et al.). Another study was conducted on 151 cucumber accessions to identify genetic loci associated with low-temperature germination (LTG). Eight candidate genes associated with abiotic stress were identified, and the function of one gene, CsPPR, was confirmed to regulate cucumber cold tolerance at the germination stage negatively. Moreover, it provides insights into cucumber LT-tolerance mechanisms and can aid in cucumber breeding development (Li et al.). Transcription factors such as MebHLH18 regulate low temperature-induced leaf abscission in cassava. MebHLH18 overexpression decreased the abscission rate, while interference expression increased it. The expression of MebHLH18 was related to POD levels and ROS scavenger levels. A single nucleotide polymorphism variation in the promoter region of MebHLH18 caused changes in its expression, leading to increased POD activity and decreased ROS accumulation, resulting in a slower leaf abscission rate at low temperatures (Liao et al.).

On the contrary, the high-temperature stress negatively affects cucumber anther, reducing pollen fertility and abnormal anther structures. The metabolites in plant hormone signal transduction and amino acid and sugar metabolism pathway were found to be associated with decreased pollen fertility. These findings provide insights into the metabolic changes in cucumber anther under HT (Chen et al.). The role of temperature is very crucial for potato storage. Higher cold conditions during potato storage can lead to the browning of chips and the development of potential carcinogens such as acrylamide during the processing of potato tubers. However, using RNAi-mediated silencing technology, there are four transgenic lines developed with reduced RS content (up to 57.5%) and acceptable chip color upon processing were obtained. The study demonstrated the efficacy of UGPase silencing in controlling CIS in potato, with potential applications for the development of CIS-tolerant potato varieties (Jaiswal et al.).

The translocation of mineral nutrients under abiotic conditions is a major factor affecting plant growth and development. A study in Chinese fir under low P conditions showed that inoculation of arbuscular mycorrhizal fungi (AMF) would promote P utilization efficiency. Low P stress conditions promoted AMF colonization and enhanced root cortex tissue dissolution, root biomass accumulation, and P use efficiency (Tian et al.). A similar kind of report was suggested by Zou et al., (2023) that arbuscular mycorrhizal fungi (AMF) affect walnut tree growth and phosphorus acquisition. They also observed that AMF directly helped with phosphorus uptake at low phosphorus levels and increased expression of certain genes involved in phosphorus transport at moderate phosphorus levels (Zou et al.). A recent report by Ihtisham et al. suggested that nitrogen, phosphorus, and potassium fertilization improved turfgrass tolerance to cold stress by enhancing antioxidant defense systems and increasing chlorophyll and carotenoid. Efficient nutrient management is important for turfgrass management in transitional climates (Ihtisham et al., 2023). The transcriptomics study in *Schima superba* in response to Mn metal stress showed 6558 DEGs. The study also identified 20 variably expressed ABC transporters in *S. superba* under Mn treatment, providing insight into the molecular mechanisms of heavy metal tolerance and detoxification in plants (Liaquat et al.). The deficiency of another essential element, such as B, can lead to affect the photosynthetic performance of sugarbeet cultivars. Song et al. indicated that B deficiency significantly impacted the growth of sugar beet leaves and the net photosynthetic rate. The study suggests that the photosynthetic rate and extent of phot-oxidative damage can be used to develop the varietal selection of sugar beet cultivars (Song et al.). Heavy metal disrupts the metabolic processes of the plant. Heavy metal such as cadmium (Cd) toxicity was reported to affect the wheat selenium-binding protein-A (TaSBP-A). TaSBP-A plays a significant role in Cd detoxification and can be used to develop Cd-tolerant plants (Luo et al.). Moreover, the role of the mineral elements such as calcium in mitigating abiotic stress was comprehensively reviewed by Feng et al.. Exogenous calcium has been shown to enhance plant resistance and tolerance to stresses through various mechanisms, including stabilizing cell walls and membranes, regulating ion ratios, maintaining photosynthesis, and inducing gene expressions and protein transcriptions (Feng et al.).

Among abiotic stress, salinity stress is a major emerging stress which affects horticultural crops. Recent reports showed that the overexpression of gene LpNAC17 from *Lilium pumilum* in transgenic tobacco resulted in increased net photosynthetic rate and chlorophyll contents and decreased stomatal conductance, transpiration rate, and intercellular CO_2_ concentration under salt stress (Wang et al.). Another study in *Lilium pumilum* showed that key genes such as NF-YB3, metallothionein type 2 protein, vicilin-like seed storage protein, and bidirectional sugar transporter SWEET14 associated with salt stress tolerance (So et al.). Similarly, another study revealed that 143 NPF genes were reported in tobacco and classified into eight subfamilies. These findings suggest that NtNPF6.13 may play a role in chloride uptake in tobacco. Additionally, several other NtNPF genes were identified as potential players in chloride metabolism, but further study is needed to confirm their roles (Zhang et al.).

Drought is detrimental to plant growth and development. Horticultural crops such as grapevine and Cynodon dactylon were reported to be affected by drought stress. The drought-responsive gene ANNEXIN (VvANN1) in grapevine showed to be induced by osmotic stress and enhances osmotic and drought tolerance by modulating the level of MDA, H_2_O_2_, and ${\text{O}}_{2}^{\cdot -}$ at the seedling stage, indicating that VvANN1 might be involved in the process of ROS homeostasis under drought or osmotic stress conditions. The control of VvANN1 might be due regulation of VvbZIP45 by controlling the expression of VvANN1 by binding in the promoter region of VvANN1 in response to drought stress (Niu et al.). On the other hand, Cynodon dactylon provides tolerance to drought stress by root modification and sclerification. Anatomical modifications that are specific to each ecotype of *Cynodon dactylon* to cope with environmental stress conditions (Tufail et al.). Another study of drought stress in C3 and C4 plant, SPEECHLESS genes may play a role in these differences, with C4 crops having evolved multiple homologs, a potential model for abiotic stress response in C3 and C4 crops (Song et al.).

The pH of soil modulates the growth and development of horticultural crops. The study on high soil pH in 15 blueberry cultivars showed variations in phenotypic and physiological. The cultivars were classified into four categories based on their tolerance level, with ‘Briteblue’ being the most tolerant and ‘Anna’ being the most sensitive. Plant height, soluble sugar, transpiration rate, leaf length, intercellular CO_2_ concentration, SOD, and SPAD were useful predictors of high soil pH tolerance (Yang et al.).

## Studies addressing factors affecting biotic stress responses in horticultural crops

Numerous studies have investigated the factors affecting biotic stress responses in horticultural crops (Khan et al.; Yadav et al.). Some of the key factors include plant genetics, environmental conditions, and cultivation practices. Plant genetics play a critical role in biotic stress responses, as the level of resistance to pests and diseases is largely determined by the plant’s genetic makeup. Breeding programs that focus on developing disease-resistant and pest-resistant cultivars have successfully enhanced the resilience of horticultural crops to biotic stress. Studies on watermelon suggest that the resistant line had higher levels of defense-related phytochromes and showed significant differences in gene expression compared to the susceptible line against powdery mildew. Most differentially expressed genes were linked to plant hormone and transduction pathways, phenylpropanoid biosynthesis, and defense responses. The study identified several potential genes, including PR1 and PRX, for further research on resistance breeding (Yadav et al.). On the contrary, Yellow Leaf Disease (YLD) affects betel palms in China region due to the velarivirus 1 (APV1). YLD symptoms worsen in winter, and severity is linked to the APV1 viral titre. Temperature also plays a role, with severe symptoms at low temperatures and moderate symptoms at high temperatures. APV1 titer is highest at low temperatures. These findings have implications for YLD epidemiology (Khan et al.). Tomato yellow leaf curl virus (TYLCV) is a harmful disease affecting tomato growth. Six genes (Ty-1 to Ty-6) have been transferred to commercial cultivars to provide protection, but only Ty-1, Ty-2, and Ty-3 are effective in some strains. This study aims to provide information on obstruction genes, sources, and indicators to help breeders develop TYLCV-resistant varieties (El-Sappah et al.).

Bacterial infection in horticultural crops might be devastating and affect the production and productivity of the crops. A recent study by Chandel et al. screened 157 onion genotypes for resistance to *Stemphylium* leaf blight (SLB) at two stages of growth. A more robust resistance breeding program can be achieved by screening at both stages and selecting genotypes with high dry matter and biochemical activity. This approach can help identify and prioritize genotypes that exhibit desirable traits related to resistance, which can ultimately lead totronger and more effective breeding strategies (Chandel et al.). The impact of galaxolide (HHCB) on the growth, physiology, and biochemistry of wheat and faba bean plants was studied by Madnay et al.. Diazotrophic plant growth-promoting *Rhodospirillum* sp. JY3 mitigated HHCB-induced stress by modulating oxidative burst, improving plant biomass and photosynthetic efficiency, augmenting antioxidants, and enhancing detoxification metabolism. Inoculation with JY3 also increased the tolerance level of both crops against HHCB contamination (Madnay et al.). Similarly, the role of the CmWRKY15-1 gene was suggested to provide resistance to White Rust disease in chrysanthemums. Silencing CmWRKY15-1 reduced the activities of antioxidant and defense enzymes and increased susceptibility to the pathogen. The gene likely increases resistance by enhancing the protective enzyme system, which could aid in breeding new resistant varieties (Chen et al.; Rahman et al.). Crop loss due to *Phytophthora sojae* in soybean results in approximately 2 billion US $ crop loss annually. Adigun et al. studied tolerant and susceptible cultivars to assess the role of phyto-oxylipin anabolism during infection. Unique oxylipin biomarkers were generated from intact oxidized lipid precursors in tolerant cultivars, while microbial-origin oxylipins were upregulated in susceptible cultivars. This study provides evidence for phyto-oxylipin metabolism in soybean cultivars during pathogen infection and its potential application in developing soybean tolerance to Phytophthora sojae (Adigun et al.).

Using biological agents is a sustainable approach to control the biotic stress in horticultural crops. The beneficial effect of endophytic bacterial strains in French marigold suggest that three endophytic bacterial strains improved growth, vase life, biochemical attributes, and antioxidant and nematocidal activities. The bacterial strains also improved the plant’s ability to scavenge radicals, resist plant-parasitic nematodes, and increase the activity of several antioxidant enzymes (Naveed et al.). Endophytic entomo-pathogenic fungi (EEPF) can be used as a potential tool for biological control. EEPF can boost plant growth, nutrition, and defense mechanisms against insect pests through changes in physio-chemical properties and the production of enzymes. IPM requires understanding the physiological mechanisms of EEPF colonization and the abiotic and biotic elements that influence plant-EEPF interaction (Samal et al.). Similarly, in another study, the combined effect of *Providencia vermicola* and iron oxide nanoparticles was observed on plant growth in Ajwain (*Trachyspermum ammi*) seedlings under different levels of arsenic contamination. However, the application of *P. vermicola* and FeO-NPs improved plant growth by decreasing arsenic content and capturing reactive oxygen species. FeO-NPs showed better results compared to *P. vermicola*. The study suggests that combining *P. vermicola* and FeO-NPs can improve plant growth and composition under metal stress (Sun et al.). *Rhizobium* is a key nitrogen-fixing microorganism that improves soil fertility and crop productivity. Using soil test-based fertilizers and adding native strains significantly increased pod yield in farmers’ fields. Both strains are recommended for the bio-inoculation of French beans(Athul et al.). The excessive use of chemical fertilizers and pesticides in agriculture causes soil problems and restricts sustainable development. *Streptomyces aureoverticillatus* HN6 is a biocontrol microorganism that improves soil fertility and controls diseases. *Streptomyces* HN6 has the potential as a biofertilizer for improving plant productivity and controlling plant pathogenic fungi (Wang et al.). A recent study suggested that *Parthenium hysterophorus*, a locally available herbaceous plant, manages the bacterial wilt of tomatoes. The *P. hysterophorus* leaf extract significantly reduced the pathogen population in soil and wilt severity on tomato plants, resulting in increased growth and yield (Najeeb and Li). In another study, *Trichoderma* spp. Isolates were obtained from the rhizospheric microflora of solanaceous horticultural crops and screened for biocontrol activity against fungal and bacterial pathogens. Molecular identification revealed that seven isolates were *T. harzianum* and one was *T. asperellum*. *In vitro* assays showed significant biocontrol activity for all eight isolates. It was concluded that *T. harzianum* MC2 and *T. harzianum* NBG show promise for use in agricultural biopesticide formulations (Rahman et al.). The suppressive effect of salicylic acid and *Cinnamomum verum* on green and blue mold in sweet oranges. The combination of SA and CV showed the lowest disease incidence and severity without affecting fruit quality. Transcriptional profiling and biochemical quantification revealed upregulation of defense enzymes associated with reduced incidence of molds. SA+CV can be a safer alternative to chemicals for post-harvest mold management (Moosa et al.).

## Conclusions and perspectives

In conclusion, the impact of abiotic and biotic stresses on horticultural crops is a significant challenge facing global food security and economies. However, recent research has shown promising strategies to enhance crop tolerance and productivity through biostimulant chemicals, microorganisms, phytochemicals, secondary metabolites, and antimicrobial peptides. Furthermore, researchers are utilizing cutting-edge technologies such as epigenomics, genomics, proteomics, and metabolomics to understand better the mechanisms involved in plant stress response and develop novel approaches to improve crop durability and output. Continuing research in this area is essential to develop sustainable and resilient horticultural crop production systems that adapt to the changing global climate and human activities. By screening for genotypes with high dry matter and biochemical activity, selecting desirable traits related to resistance, and utilizing the latest technologies and strategies, researchers can help develop more robust and effective breeding programs. Ultimately, these efforts have the potential to significantly enhance crop plant tolerance to stress and contribute to the production of high-quality, nutritious, and sustainable food for the growing global population.

## Author contributions

All authors listed have made a substantial, direct, and intellectual contribution to the work and approved it for publication.
